# Use of oral contraceptives in *BRCA* mutation carriers and risk for ovarian and breast cancer: a systematic review

**DOI:** 10.1007/s00404-020-05458-w

**Published:** 2020-03-05

**Authors:** D. Huber, S. Seitz, K. Kast, G. Emons, O. Ortmann

**Affiliations:** 1 Department of Gynecology and Obstetrics, University Medical Center Regensburg, Regensburg, Germany; 2 Department of Gynecology and Obstetrics, Medical Faculty and University Hospital Carl Gustav Carus, TU Dresden, Dresden, Germany; 3 Department of Gynecology and Obstetrics, Georg August University Göttingen, University Medicine, Göttingen, Germany

**Keywords:** Oral contraception, Breast cancer, Ovarian cancer, *BRCA1*, *BRCA2*

## Abstract

**Purpose:**

*BRCA* mutation carriers have an increased risk of developing breast or ovarian cancer. Oral contraception (OC) is known to increase breast cancer and reduce ovarian cancer risk in the general population. This review analyses the published data on OC and risk of cancer in *BRCA* mutation carriers.

**Methods:**

We included all relevant articles published in English from 1995 to 2018. Literature was identified through a search on PubMed and Cochrane Library.

**Results:**

We included four meta-analyses, one review, one case–control study and one retrospective cohort study on the association between ovarian cancer and OC in *BRCA* mutation carriers. All report a risk reduction for the OC users and several also describe an inverse correlation with duration of use. Regarding breast cancer, we included four meta-analyses, one review, one case–control study, two case-only studies, one prospective and one retrospective cohort study. Some studies report a risk elevation, while others did not find an association between OC use and breast cancer in *BRCA* mutation carriers. In other studies, the association was limited to early-onset breast cancer and/or associated with young age at first start of OC.

**Conclusion:**

Oral contraception leads to a risk reduction of ovarian cancer also in *BRCA* mutation carriers. An increase in breast cancer risk due to OC cannot be excluded. Women with BRCA mutation who consider OC use have to be informed about possible increase in breast cancer risk and alternative contraceptive methods. OC should not be used for the prevention of ovarian cancer in this population.

## Introduction

Mutations in *BRCA1/2* genes represent significant risk factors for breast and ovarian cancer. A recent prospective study suggests a cumulative risk to the age of 80 years in *BRCA1* mutation carriers of up to 72% for breast cancer and up to 44% for ovarian cancer. In *BRCA2* mutation carriers, the cumulative breast cancer risk to the age of 80 years according to this data is increased up to 69% and for ovarian cancer up to 17%, respectively [1].

The estimated prevalence of mutations in *BRCA1* and *2* genes varies between 0.3 and 0.8%. Depending on the investigated population, the prevalence of founder mutations can be significantly higher [2]. Besides the genetic risk factors, there are other risk modifying factors, e.g. endocrine interventions. Among these, one of the most common is oral contraception (OC). In the general population, current or recent use of oral contraceptives leads to an increased risk for breast cancer (RR = 1.20; 95% CI 1.14–1.2). The risk caused by combined oral contraceptives appears to vary depending on the duration of use and the type of progestin [3]. In contrast, use of OC leads to a profound decrease of ovarian cancer risk by 20% RR reduction for every 5 years of use. This effect persists for many years after cessation of use [4]. There is little data on the combined effects of various risk factors, such as obesity, exercise or genetic mutations. In this review, we analyse the published data on OC and risk for breast or ovarian cancer in *BRCA1/2* mutation carriers.

## Methods

A search for relevant articles was run from 1995 to 2018 on PubMed. The MeSH used for the search were “oral contraceptives” and “BRCA mutation” and “oral contraceptives” and “breast cancer” or “ovarian cancer”. The search created 59, 1634 and 1146 hits, respectively. We also conducted a search within the Cochrane library. There were no Cochrane reviews available on OC and risk of cancer in BRCA1/2 mutation carriers. The hits were searched for relevance, clinical trials, reviews and meta-analyses. Also, the references of appropriate articles were screened for relevant publications. The authors of this review defined all those articles which deal with the specific subject of whether or not OC has an impact on breast and/or ovarian cancer in *BRCA1/2* mutation carriers as relevant.

We found 31 relevant publications, 7 of which were regarding both cancer risks, 8 regarding the risk of ovarian cancer and 16 regarding breast cancer risk only. After excluding cross-match, i.e. excluding publications which were part of an included review or meta-analysis, there remained a total of 13 publications to include in our review. Among those, there were four meta-analyses and one systematic review regarding both ovarian and breast cancer risk. Furthermore, we included one case–control study and one retrospective cohort study on ovarian cancer risk and one case–control, two case-only studies, one prospective and one retrospective cohort study on OC-induced breast cancer risk in *BRCA* mutation carriers. Due to differences in populations, study types, statistical methodology and inclusion criteria, the studies included in this review were not appropriate for meta-analysis.

## Results

### Ovarian cancer

After excluding cross-match, we included seven publications on ovarian cancer risk associated with OC use in *BRCA* mutation carriers. Among those, there were four meta-analyses, one review, one case–control study and one retrospective cohort study. All of them report a risk reduction for the OC users and several also describe an inverse correlation with ovarian cancer risk and duration of OC use (Table 1).

**Table 1:** Oral contraception and risk of ovarian cancer in BRCA mutation carriers

Study/study design/Oxford Center of Evidence-based Medicine (OCEBM) level of evidence (LOE)	Number	Results
Narod et al. [5] Case–control study LOE 3b	Cases: 179 *BRCA1*, 28 *BRCA2* Controls: 161 (50 *BRCA1*, 3 *BRCA2*, 42 without mutation, 66 without testing)	Risk reduction *BRCA1*: OR = 0.5; 95% CI 0.3–0.9 *BRCA2*: OR = 0.4; 95% CI 0.2–1.1 Combined: OR = 0.5; 95% CI 0.3–0.8 Inverse correlation with duration of OC (*p* trend < 0.001)
Iodice et al. [6] Meta-analysis LOE 2a Included studies Four case–control studies [7, 9, 10, 14] One retrospective cohort study [8]	Cases: 1262 *BRCA1*, 253 *BRCA2*, 1 *BRCA1 *+ *2* Controls: 2678 *BRCA1*, 538 *BRCA2*	Risk reduction *BRCA1*: SRR = 0.51; 95% CI 0.40–0.6 *BRCA2*: SRR = 0.50; 95% CI 0.29–0.89 (*p* = 0.88) Combined: SRR = 0.50; 95% CI 0.33–0.75 Linear decrease in risk of 36%/10 years (95% CI 22–47%, *p* trend < 0.01)
Cibula et al. [11] Review LOE 2a Included studies Five case–control studies [7, 10, 12, 25, 38] One retrospective cohort study [8]	Cases: 1203 *BRCA1*, 277 *BRCA2*, 1 *BRCA1 *+ *2*, 282 *BRCA1/2* (not further indicated, from [25]) Controls: 2160 *BRCA1*, 432 *BRCA2*	Risk reduction in all but one [12] of the included studies Partly inverse correlation with duration of OC
Cibula et al. [13] Meta-analysis LOE 2a Included studies Three case–control studies [7, 10, 14]	Cases: 934 *BRCA1*, 161 *BRCA2*, 1 *BRCA1 *+ *2* Controls: 2307 *BRCA1*, 413 *BRCA2*	Risk reduction *BRCA1*: OR = 0.56; 95% CI 0.49–0.69 (*p* < 0.001) *BRCA2*: OR = 0.49, 95% CI 0.32–0.77 (*p* < 0.002) Combined: OR = 0.57; 95% CI 0.47–0.70 (*p* < 0.001) Inverse correlation with duration of OC: OR = 0.95; 95% CI 0.93–0.97 (*p* < 0.001)
Moorman et al. [15] Meta-analysis LOE 2a Included studies Five case–control studies [7, 10, 12, 14, 38] One retrospective cohort study [8]	Cases: 1353 *BRCA1*, 277 *BRCA2* Controls: 2310 *BRCA1*, 423 *BRCA2*	Risk reduction *BRCA1*: OR = 0.55; 95% CI 0.47–0.66 (*p* = 0.743) *BRCA2*: OR = 0.65; 95% CI 0.34–1.24 (*p* = 0.096) Combined: OR = 0.58; 95% CI 0.46–0.73 (*p* = 0.210)
Friebel et al. [16] Review and meta-analysis LOE 2a Included studies Four case–control studies [10, 14, 17, 18] One retrospective cohort study [8]	Cases: 1348 *BRCA1*, 239 *BRCA2*, 1 *BRCA1 *+ *2* Controls: 2926 *BRCA1*, 439 *BRCA2*	Risk reduction *BRCA1*: Risk reduction associated with use. Risk reduction of 33–80% for OC use > 1 year *BRCA2*: Risk reduction of 58–63% associated with use
Perri et al. [19] Retrospective cohort study LOE 2b	Cases: 139 *BRCA1*, 33 *BRCA2*, 3 unknown Controls: 579 *BRCA1*, 298 *BRCA2*, 3 *BRCA1 *+ *2*, 19 unknown	Risk reduction *BRCA1*: OR = 0.21; 95% CI 0.14–0.33 (*p* < 0.001) *BRCA2*: OR = 0.24; 95% CI 0.09–0.61 (*p* < 0.001) Combined: OR = 0.19; 95% CI 0.13–0.28 (*p* < 0.001) Inverse correlation with duration of OC

A case–control study from 1998 by Narod et al. [5] found a risk reduction associated with ever use compared to never use of OC. The risk reduction was present in *BRCA1* (OR = 0.5; 95% CI 0.3–0.9) and *BRCA2* (OR = 0.4; 95% CI 0.2–1.1) mutation carriers, with a limited number of BRCA2 mutation carriers included in the study. There was an inverse correlation with ovarian cancer risk and duration of OC (*p* for trend < 0.001), with a risk reduction of 60% for a duration of OC use for 6 years and more. The average duration of OC was 4 years for cases and 6 years for control women. The mean age of beginning OC use was 24 years for cases and 22 years for controls. The controls were living sisters of the patients and they were included whether or not information on molecular testing was available. Also, 30% of the patients and 18% of the controls had a history of breast cancer and more than one-third of the controls had undergone bilateral oophorectomy before enrollment. Specific ethnic groups were slightly overrepresented, that is Ashkenazi Jewish within the cases and French-Canadian women within the control group.

A study by Iodice et al. [6] from 2010 included four case–control studies and one retrospective cohort study. The study included 1262 cases and 2678 controls with *BRCA1* mutation, 253 cases and 538 controls with *BRCA2* mutation and 1 case with *BRCA1* and *2* mutation. A meta-analysis confirmed significantly reduced risk for ovarian cancer for BRCA1 (SRR = 0.51; 95% CI 0.40–0.65) and *BRCA2* mutations carriers (SRR = 0.50; 95% CI 0.29–0.89) associated with use of OC. Also, increasing duration of OC use was associated with a linear decrease in risk of 36% for each additional 10 years (95% CI 22–47%; *p* < 0.01). The definition for use of OC was > 1 year in one of the included case–control study [7] and any duration of use in the rest of the studies. The mean age at enrollment varied between 41 [8] and 53 years [9, 10]. Analysis of age at the beginning of use and its association with ovarian cancer risk was not performed. All of the included studies were retrospective and their designs partly differed.

A review by Cibula et al. [11] included five case–control studies and one retrospective cohort study. The included studies comprise a total of 1203 cases and 2160 controls with *BRCA1* mutation, 277 cases and 432 controls with BRCA2 mutation, 1 case with *BRCA1* and *2* mutation and 282 cases with not further indicated BRCA1/2 mutation carriers. Three of the included studies [7, 8, 10] were included in the meta-analysis by Iodice [6]. Of the five studies included in the review, only one did not confirm a protective effect for ovarian cancer in *BRCA* mutation carriers (OR for 5 or more years of OC use = 1.07; 95% CI 0.63–1.83) [12]. The review provides the specific ethnic/Jewish background and the small number of OC users as possible explanations. The five other studies showed a decrease of ovarian cancer risk associated with OC use. In some of the studies, the protective effect was associated with duration of OC use of at least 1 year [7, 12]. One of the included studies found a protective effect associated with use of OC, restricted to *BRCA1* mutation carriers (HR = 0.52; 95% CI 0.37–0.73; *p* = 0.0002) [8]. Some of the studies included only *BRCA1* mutation carriers or a restricted number of *BRCA2* mutation carriers. The largest study included 1 ovarian cancer case with *BRCA1* and *2* mutation, 670 ovarian cancer cases and 2043 controls with BRCA1 mutation as well as 128 cases and 380 controls with BRCA2 mutation [10]. It confirmed a risk reduction for both BRCA1 (OR = 0.56; 95% CI 0.45–0.71, *p *< 0.0001) and *BRCA2* mutation carriers (OR = 0.39; 95% CI 0.23–0.66, *p* = 0.0004) associated with use of OC. Also, a significant trend of risk reduction associated with increasing duration of use was reported (*p* < 0.0001) in this study. Not all of the studies included in the review provided molecular testing of all controls. All of the studies were retrospective and the designs were different.

A meta-analysis by the same author confirms a protective effect for any past use of OC and a trend in risk with increasing duration of OC use [13]. It includes three case–control studies, one of which [14] included only *BRCA1* mutation carriers. In the meta-analysis, the protective effect was shown for BRCA1 (OR = 0.56; 95% CI 0.49–0.69; *p* < 0.001) as well as BRCA2 mutation carriers (OR = 0.49, 95% CI 0.32–0.77; *p *< 0.002). The OR for pooled trend in risk with increasing duration of OC use was 0.95 (95% CI 0.93–0.97; *p* < 0.001). The age at start of use and its association with ovarian cancer risk was not studied, neither were type and dosage of OC.

The most recent meta-analysis by Moorman et al. is also the largest of the included studies, considering 1353 ovarian cancer cases and 2310 controls with *BRCA1* mutation as well as 277 cases and 423 controls with *BRCA2* mutation [15]. Comparing ever use of and never use of OC, the OR for *BRCA1* mutation carriers was 0.55 (95% CI 0.47–0.66). The OR for *BRCA2* mutation carriers was 0.65 (95% CI 0.34–1.24), the difference between the two groups was not statistically significant (*p* = 0.975). All of the included studies demonstrated an inverse association between ovarian cancer risk and duration of OC use. However, these findings could not be used for the meta-analysis due to differences in duration categories. Only one of the studies reported an effect by time since last OC use, with a lower risk for more recent users (< 10 years) [8]. As none of the studies listed type and dose of contraceptive pills, the possible effect of different combinations and dosage of OC could not be evaluated. Mean age at OC start and duration was not reported. All of the included studies were observational and some did not exclude prevalent cancer cases.

Friebel et al. [16] conducted a systematic review and meta-analysis on modifiers of cancer risk in BRCA 1 and 2 carriers. Regarding OC in *BRCA* mutation carriers, they included four case–control studies and one retrospective cohort study with a total of 1348 cases and 2926 controls with *BRCA 1* mutation, 239 cases and 439 controls with *BRCA 2* mutation and 1 case with *BRCA1 *+* 2* mutation. For *BRCA1* mutation carriers, four of the included studies reported a risk reduction of ovarian cancer associated with use of OC [8, 10, 14, 17]. One of the studies showed no association between OC and ovarian cancer risk [18]. Regarding the duration of OC use, all of the included studies that examined OC use > 1 year showed a statistically significant risk reduction from 33 to 80% in *BRCA1* mutation carriers [8, 10, 14]. Due to overlapping samples, no meta-analysis could be performed. For *BRCA2* mutation carriers, two of the included studies reported a risk reduction from 58 to 63% associated with use of OC [10, 17]. Data were not sufficient to perform a meta-analysis in *BRCA2* mutation carriers and the duration of use was not further examined. Age at start of use or the type of OC were not reported.

Perri et al. [19] conducted a study on ovarian cancer risk for *BRCA* mutation carriers undergoing fertility treatment. All of the participants were Jewish Israeli women with personal or family history of BRCA mutation-associated cancers. A multivariate analysis showed a reduced risk for *BRCA1* (OR = 0.21; 95% CI 0.14–0.33) and *BRCA2* mutation carriers (OR = 0.21; 95% CI 0.09–0.61) associated with use of OC. There was a further risk reduction with duration of use: the OR for up to 1 year of OC use was 0.36 (95% CI 0.16–0.84), for more than 5 years 0.10 (95% CI 0.06–0.17). Women who had undergone risk-reducing oophorectomy were not excluded from the study. The mean age was 53.6 years for cases and 49.1 for controls. There was no information on age at start of use, duration or type of OC.

**Table 2:** Oral contraception and risk of breast cancer in BRCA mutation carriers

Study/study design/Oxford Center of Evidence-based Medicine (OCEBM) level of evidence (LOE)	Number	Results
Pasanisi et al. [20] Case-only study LOE 4	382 “genetic”, 1333 “sporadic” cases	Borderline significant association Genetic cases: OR = 1.3; 95% CI 1.0–1.7 (*p* = 0.05) Highest association for OC start at 18–20 years: OR = 1.6; 95% CI 1.1–2.3 (*p* trend = 0.18) Duration of use not statistically significant (*p* = 0.32)
Iodice et al. [6] Meta-analysis LOE 2a Included studies Four case–control studies [14, 21, 25, 28] One retrospective cohort study [26]	Cases: 2154 *BRCA1*, 707 *BRCA2* Controls: 2280 *BRCA1*, 672 *BRCA2*	No significant association *BRCA1*: RR = 1.09; 95% CI 0.77–1.54 *BRCA2*: RR = 1.15; 95% CI 0.61–2.18 Combined: SRR = 1.33; 95% CI 0.88–1.45) No association with duration of use (*p* = 0,2)
Cibula et al. [11] Review LOE 2a Included studies Seven case–control studies [21, 25, 27, 28, 39–41] One retrospective cohort study [26]	Cases: 2151 *BRCA1*, 862 *BRCA2*, 94 *BRCA1/2* (not further indicated, from [40]) Controls: 2121 *BRCA1*, 719 *BRCA2*	Mild to moderate increase in risk Further increase in risk when OC duration ≥ 4 years before FFT (BRCA1: HR = 1.49; 95% CI 1.05–2.11. BRCA 2: HR = 2.58; 95% CI 1.21–5.49)
Cibula et al. [13] Meta-analysis LOE 2a Included studies Three case–control studies [14, 25, 28] Two retrospective cohort studies [21, 26] Five case–case studies [23, 24, 27, 40, 42]	Case–control studies: Cases: 1524 *BRCA1*, 458 *BRCA2* Controls: 1631 *BRCA1*, 509 *BRCA2*	No significant association *BRCA1*: OR = 1.08; 95% CI 0.94–1.25 (*p* = 0.25) *BRCA2*: OR = 1.03; 95% CI 0.81–1.32 (*p* = 0.788)
	Cohort studies: Cases: 630 *BRCA1*, 249 *BRCA2* Controls: 649 *BRCA1*, 163 *BRCA2*	Increase in risk *BRCA1*: OR = 1.48; 95% CI 1.14–1.92 (*p* = 0.727)
	Case–case studies: 131 *BRCA1*, 80 *BRCA1*, 1 *BRCA1 *+ *2*, 94 *BRCA1/2* (not further indicated, from [40])	No increase in risk *BRCA1/2*: OR = 0.80; 95% CI 0.59–1.08 (*p* = 0.147)
Moorman et al. [15] Meta-analysis LOE 2a Included studies Three case–control studies [14, 25, 28] Two retrospective cohort studies [26, 29]	Cases: 2401 *BRCA1*, 830 *BRCA2* Controls: 2215 *BRCA1*, 672 *BRCA2*, 373 *BRCA1/2* (not further indicated, from [29])	Non-statistically relevant increase in risk *BRCA1*: OR = 1.19; 95% CI 0.92–1.55 (*p* = 0.004) *BRCA2*: OR = 1.36; 95% CI 0.89–2.10 (*p* = 0.022) Combined: OR = 1.21; 95% CI 0.93–1.58 (p < 0.001)
Kotsopoulos et al. [30] Case–control study LOE 3b	2492 *BRCA1* case–control pairs	Increase in risk when starting < 20 years: OR = 1.45; 95% CI 1.20–1.75 (*p* = 0.0001) No statistically relevant increase in risk when starting at 20–25 years: OR = 1.19; 95% CI 0.99–1.42 (*p* = 0.06) Effect only for early-onset < 40 years: OR = 1.40; 95% CI 1.14–1.70 (*p* = 0.001)
Friebel et al. [16] Review and meta-analysis LOE 2a Included studies: Five case–control studies: [14, 21, 25, 28, 32] Two retrospective cohort studies [26, 29]	Case–control studies: Cases: 3606 *BRCA1*, 1257 *BRCA2* Controls: 3730 *BRCA1*, 1308 *BRCA2*	No association *BRCA1*: ES = 0.78; 95% CI 0.59–1.04 *BRCA2*: ES = 1.04; 95% CI 0.81–1.32 [25, 28]
	Cohort studies: Cases: 877 *BRCA1*, 372 *BRCA2* Controls: 584 *BRCA1*, 163 *BRCA2*, 373 *BRCA1/2* (not further indicated, from [29])	Increase in risk *BRCA1*: ES = 1.59; 95% CI 1.32–1.92 *BRCA2*: ES = 1.85; 95% CI 1.30–2.64 No association with duration of use
Rieder et al. [33] Case-only study LOE 4	258 *BRCA1*, 108 *BRCA2*	Prior or current OC associated with younger age at diagnosis: HR = 1.7; 95% CI 1.1–2.05 (*p* = 0.006) No association with duration of use: HR = 1.00; 95% CI 0.99–1.00
Park et al. [34] Retrospective cohort study LOE 2b	Cases: 168 *BRCA1*, 109 *BRCA2* Controls: 54 *BRCA1*, 250 *BRCA2*	No significant association *BRCA1*: HR = 1.24; 95% CI 0.45–3.40 *BRCA2*: HR = 0.71; 95% CI 0.21–2.37
Schrijver et al. [35] Retrospective and prospective cohort study LOE 1b	Prospective cohort: Cases: 269 *BRCA1*, 157 *BRCA2* Controls: 2007 *BRCA1*, 1453 *BRCA2*	No association for *BRCA1* (HR = 1.08; 95% CI 0.75–1.5), increase in risk for *BRCA2* (HR = 1.75; 95% CI 1.03–2.9)
	Retrospective cohort, left-truncated: Cases: 1095 *BRCA1*, 752 *BRCA2* Controls: 2733 *BRCA1*, 1760 *BRCA2*	Increase in risk for *BRCA1* (HR = 1.26; 95% CI 1.06–1.51), no association for *BRCA2* (1.06; 95% CI 0.85–1.33)
	Retrospective full-cohort: Cases: 2525 *BRCA1*, 1548 *BRCA2* Controls: 3180 *BRCA1*, 1973 *BRCA2*	Increase in risk for *BRCA1* (HR = 1.39; 95% CI 1.23–1.58) and *BRCA2* (HR = 1.52; 95% CI 1.28–1.81)
		Inverse correlation with duration of use, especially before FFTP (*BRCA1*: both retrospective analyses, *p* < 0.001 and *p* = 0.001; *BRCA2*: full retrospective analysis, *p* = 0.002)

### Breast cancer

The included studies on the association between OC use and breast cancer risk in *BRCA* mutation carriers comprise four meta-analyses, one review, one case–control-study, two case-only studies, one retrospective and one prospective cohort study. Some of the studies report a risk elevation, while others did not find an interaction between OC use and breast cancer (Table 2).

Pasanisi et al. [20] conducted a case-only study which resulted in a borderline significant association between genetic breast cancer and OC use compared with never use (OR = 1.3; 95% CI 1.0–1.7). The highest association was found for OC start between 18 and 20 years (OR, 1.6; 95% CI 1.1–2.3). From the results, the research group inferred a higher vulnerability to OC for women with *BRCA* mutation. Women with breast cancer before the age of 45 years who were then classified as sporadic or genetic cases were included. There was no systematic genetic testing and classification of genetic cases was based on software-assisted mathematical *BRCA* mutation probability. The duration of OC use was analysed as well. However, the comparison between duration of more than 5 years and a shorter duration of maximum 5 years of use was not statistically significant. There was no information on type and dosage of OC.

In the meta-analysis by Iodice et al. [6], not only the impact of OC on breast cancer, but also on ovarian cancer risk in *BRCA* mutation carriers was examined. A minority of the included women had a history of ovarian cancer [21]. The meta-analysis included four case–control studies and one retrospective cohort study. The study found no significant association between OC and breast cancer risk for *BRCA1* (RR = 1.09; 95% CI 0.77–1.54) and *BRCA2* mutation carriers (RR = 1.15; 95% CI 0.61–2.18). An association between duration of use was not found, either. A significant increased risk was found for OC formulations before 1975 (RR 1.47; 95% CI 1.06–2.04), but not for more recent preparations (RR: 1.17; 95% CI 0.74–1.86). The mean age of women varied between 33 [22] and 45 years [23]. The definition of OC use was more than 1 year in one [24] and any duration of use in the rest of the included studies. All of them were retrospective and the study designs were not identical. Some included controls even if molecular testing was not available. Genetic testing of the enrolled controls in the metanalysis was therefore not complete.

The large review conducted by Cibula et al. [11] from 2010 included a total of seven case–control and one retrospective cohort study. The studies mostly found a mild or moderate risk elevation, but the power was low. The largest study included 981 case–control pairs with *BRCA1* and 330 pairs with *BRCA2* mutations [25]. An elevated risk associated with OC use was found only for BRCA1 mutation carriers with an early diagnosis before the age of 40 years (OR = 1.38; 95% CI 1.11–1.72). An elevated risk was not detected for *BRCA1/2* mutation carriers who developed breast cancer after the age of 40 years. The second largest study included a cohort of 1181 *BRCA1* and 412 *BRCA2* mutation carriers [26]. Of those, 597 *BRCA1* and 249 *BRCA2* mutation carriers were diagnosed with breast cancer. An elevated risk associated with OC use was found for both *BRCA1* (HR = 1.47; 95% CI 1.13–1.91) and *BRCA2* mutation carriers (HR = 1.49; 95% CI 0.82–2.70). A further increase in risk was seen when the duration of OC use was at least 4 years before the first full-term pregnancy (FFTP); the observed HRs were not significantly different from those in women without family history (BRCA1: HR = 1.49; 95% CI 1.05–2.11. BRCA 2: HR = 2.58; 95% CI 1.21–5.49) [26]. The included studies were all retrospective. Study designs were not identical and some of the studies matched cases with controls without mutation [27, 28]. The authors conclude that OC use might be associated with a weak risk elevation for breast cancer in patients with *BRCA* mutation, but the risk–benefit balance is influenced positively by the protective effect on ovarian cancer.

In their meta-analysis from 2011, Cibula et al. [13] also examined the association of OC with breast cancer in *BRCA* mutation carriers. Three case–control, two retrospective cohort and five case–case studies were included. 27 of the *BRCA1* mutation carriers, either cases or controls, had a history of ovarian cancer [21]. A meta-analysis was carried out separately for the different study types. In case–control studies, inverse variance pooling did not find a relevant risk elevation associated with OC for *BRCA1* (OR = 1.08; 95% CI 0.94–1.25) and *BRCA2* (OR = 1.03; 95% CI 0.81 1.32). Likewise, no elevation of risk was found for *BRCA1/2* mutation carriers in case–case studies (OR = 0.80; 95% CI 0.59–1.08). However, a meta-analysis of the cohort studies showed a significant increase in risk associated with OC use for BRCA1 mutation carriers (OR = 1.48; 95% CI 1.14–1.92). This result was mainly driven by one study which represented 98% weight of the data sample set [26]. Exposure to OC was defined as use for more than 1 year in one case–control and one case–case study, use for more than 3 months in one cohort study and as long-term use in one case–case study [21, 24, 27, 28]. In all of the other studies, OC exposure was defined as use. A trend in risk with duration of use could not be evaluated due to different populations and study designs. Information on type and dosage of OC was not given. Overall, the included studies showed inconsistent results and many of them had a limited group size.

In their meta-analysis, Moorman et al. [15] included three case–control studies and two retrospective cohort studies. The case–control [14, 25, 28] and one of the cohort studies [26] are identical with those in the meta-analysis by Cibula et al. in 2011 [13]. The authors found a risk elevation associated with OC use for *BRCA1* (OR = 1.19; 95% CI 0.92–1.55) and *BRCA2* mutation carriers (OR 1.36; 95% CI 0.89–2.10) which was not statistically significant. They draw the conclusion that the association between breast cancer and use of OC among *BRCA* mutation carriers does not differ greatly from the general population. The duration and timing of use could not be further examined due to inadequate data. A possible effect of type and dosage of OC could not be examined, either. The included studies were all observational and study designs were not identical. Some of the studies did not exclude prevalent cancer cases and some included patients from specific ethnic subgroups, such as Ashkenazi Jewish and Polish women [14, 29].

Kotsopoulos et al. included 2492 case–control pairs with *BRCA1* mutation. They found an increase in breast cancer risk for *BRCA1* mutation carriers who started OC before the age of 20 years (OR = 1.45; 95% CI 1.20–1.75; *p* = 0.0001) and a non-significant increase for carriers who started between 20 and 25 years of age (OR 1.19; 95% CI 0.99–1.42; *p* = 0.06) [30]. When adjusted for age at diagnosis, the effect was observed only for early-onset breast cancer with diagnosis before the age of 40 years (OR = 1.40; 95% CI 1.14–1.70; *p* = 0.001). When breast cancer was diagnosed at or after 40 years of age, no increase in risk was reported (OR 0.97; 95% CI 0.79–1.20; *p* = 0.81). The association between use of OC and early-onset breast cancer was strongest for women who started OC before the age of 20 years (OR = 1.74; 95% CI 1.36–2.22; *p* = 0.00001). An increase in risk of early-onset breast cancer was also observed when the age at OC start was between 20 and 25 years (OR = 1.36, 1.07–1.73; *p* = 0.02). The mean age at diagnosis in the study was 39.7 years, the mean age at recruitment 46.3 years. The mean duration of use was 3.8 for cases and 3.5 years for controls. The association between breast cancer risk and time since last OC use was also examined. Compared with never use, the study found no association between current OC use and breast cancer risk (OR = 0.8; 95% CI 0.66–0.97). 5 or more years after stopping OC use, a significant increase in risk of 38% was observed (OR = 1.38; 95% CI 1.18–1.61). Information on type and dosage of OC was not provided.

Friebel et al. [16] included five case–control and two retrospective cohort studies in their review and meta-analysis regarding OC use and breast cancer risk in *BRCA* mutation carriers. Two of the studies included only *BRCA1* mutation carriers [21, 31]. In total, the included studies comprised 4483 cases and 4314 controls with *BRCA1* mutation, 1629 cases and 1471 controls with *BRCA2* mutation as well as 373 controls with no further indicated *BRCA 1/2* mutation. For *BRCA1* mutation carriers, the meta-analysis of the case–control studies did not find an association between use of OC and breast cancer risk (ES = 0.78; 95% CI 0.59–1.04). The meta-analysis of the cohort studies, however, showed an increase in breast cancer risk associated with use of OC in *BRCA1* mutation carriers (ES = 1.59; 95% CI 1.32–1.92). The duration of use was also examined, but the meta-analysis did not find an effect when subcategorized in durations of > 1 year, 1–3 years and more than 3 years. For *BRCA2* mutation carriers, a meta-analysis of two case–control studies showed no association with use of OC (ES = 1.04; 95% CI 0.81–1.32) [25, 28]. The two case–control studies used for the meta-analysis comprised 458 cases and 509 controls with *BRCA2* mutation. A meta-analysis of the cohort studies, however, found an association between use of OC and breast cancer risk in *BRCA2* mutation carriers (ES = 1.85; 95% CI 1.30–2.64). Two of the case–control studies were used to examine a possible effect of the duration of use [28, 32]. No association was found for a duration of 1–3 years and more than 3 years compared to never use in *BRCA2* mutation carriers. There was no information on type and dosage of OC.

In a recent case-only study by Rieder et al. [33], multivariate analysis found an association between prior or current OC use and a younger age at diagnosis in *BRCA1/2* mutation carriers (HR = 1.7; 95% CI 1.1–2.05; *p* = 0.006). The study included 258 *BRCA1* and 108 *BRCA2* mutation carriers with a history of breast cancer. The median year of birth in the study population was 1965. Median age at diagnosis was 58 for women who were born earlier and 42 years for women who were born in or after 1965. The authors paralleled these findings with the fact that in the later birth cohort, the probability of having experienced pregnancies was lower and OC use more likely. No association was found between breast cancer onset and duration of OC (HR = 1.00; 95% CI 0.99–1.00) or starting age (HR = 1.03; 95% CI 0.8–1.3). Differences in type or dosage of OC were not described.

Park et al. [34] recruited 581 *BRCA* mutation carriers for a retrospective cohort study. The study included 222 *BRCA1* mutation carriers, 168 of which with a history of breast cancer, and 359 *BRCA2* mutation carriers, 109 with a history of breast cancer, respectively. Also, the study included non-carriers with positive family history or other high-risk criteria. Use of OC was associated with breast cancer only for non-carriers (HR = 3.99, 95% CI 1.65–9.67), but no association was found for *BRCA1* (HR = 1.24; 95% CI 0.45–3.40) and *BRCA2* mutation carriers (HR = 0.71; 95% CI 0.21–2.37). Type and dosage of OC as well as duration of use or age at first start were not examined. Study design was retrospective and the study population consisted of Asian BRCA mutation carriers.

In the most recent study, Schrijver et al. [35] included a total of 6030 *BRCA1* and 3809 *BRCA2* mutation carriers to perform prospective, left-truncated retrospective and full-cohort retrospective analyses. Women in the prospective cohort had no history of cancer or risk-reducing mastectomy at the time of inclusion. Follow-up started at birth in the full-cohort retrospective analysis. The left-truncated cohort included only BRCA mutation carriers without a history of cancer or risk-reducing mastectomy at the start of follow-up, 5 years preceding the baseline questionnaire.

For *BRCA1* mutation carriers, the prospective analysis found no association between use of OC and breast cancer risk (HR = 1.08; 95% CI 0.75–1.5). No association was found with the total duration of use, age at first use, recency of use or duration of use before FFTP in the prospective cohort. In contrast, both the left-truncated (HR = 1.26; 95% CI 1.06–1.51) and the full-cohort retrospective analysis (HR = 1.39; 95% CI 1.23–1.58) found an increase in risk associated with use of OC in *BRCA1* mutation carriers. There was an inverse correlation between increase in risk and lifetime duration of use (*p* trend = 0.01) as well as duration of use before FFTP (*p* trend = 0.001) in both retrospective cohorts. When stratified by age, the left-truncated cohort analysis indicated that the trend associated with OC use before FFTP was restricted to women at the age of ≤ 35 years (*p* difference = 0.08). Additionally, an increased risk with younger age at first OC use (*p* trend < 0.01) and longer duration of use after FFTP (*p* trend = 0.02) was observed in the full-cohort, but not in the left-truncated cohort. Stratification by age was not possible in the prospective cohort due to small sample size.

For *BRCA2* mutation carriers, the prospective analysis showed an increase in risk associated with use of OC (HR = 1.75; 95% CI 1.03–2.9). The results of the retrospective analyses were inconsistent: the left-truncated analysis found no association (HR = 1.06; 95% CI 0.85–1.33), while the full-cohort retrospective analysis showed an increase in breast cancer risk associated with use of OC (HR = 1.52; 95% CI 1.28–1.81). An increased risk for women with younger age at first use (*p* trend < 0.01) and an association with longer duration of use (*p* trend = 0.001), especially for use before FFTP (*p* trend = 0.002), were observed in the full-cohort, but not in the left-truncated and the prospective cohort.

Possible explanations for the inconsistencies between the prospective and the retrospective analyses provided by the authors were a survival bias or an underrepresentation of young women in the prospective cohort. In this cohort, the proportion of young women with breast cancer before the age of 35 years was lowest for both *BRCA1* and *BRCA2* mutation carriers (14.1 and 8.2%, respectively). Furthermore, the power of the prospective analysis might have been too low to detect trends associated with duration of use or starting age. The authors conclude that the safety of long-term OC use in *BRCA* mutation carriers remains uncertain.

## Discussion

For ovarian cancer, current data confirm a risk reduction associated with use of OC in *BRCA* mutation carriers. Several studies also describe an inverse correlation with the duration of OC use [5, 6, 11, 13, 19].

Data on breast cancer risk associated with OC use in *BRCA* mutation carriers are heterogeneous. Some of the studies report an increase in risk associated with use of OC [11, 13], while others did not find an association between breast cancer risk and current preparations of OC [6, 13, 15, 34]. In some studies, the association was limited to early-onset breast cancer with diagnosis before 40 years of age and/or associated with young age of under 20 years at first start of OC [20, 30, 33]. Some of the studies examined the duration of OC use and showed no significant association with breast cancer risk [6, 20, 33]. The review by Cibula which showed a mild to moderate increase in breast cancer risk associated with use of OC showed a further increase in risk when the duration of OC use was at least 4 years before FFTP; the observed HR was comparable with that in women without family history (*BRCA1*: HR = 1.49; 95% CI 1.05–2.11. *BRCA 2*: HR = 2.58; 95% CI 1.21–5.49) [11]. The most recent publication by Schrijver et al. [35] showed similar results. The study found an increase in risk for *BRCA1* mutation carriers associated with use of OC in both the left-truncated and the full-cohort retrospective analysis and an inverse correlation with duration of use before FFTP which, however, was restricted to women at the age of ≤ 35 years in the left-truncated retrospective cohort (*p* difference = 0.08). For *BRCA2* mutation carriers, a retrospective analysis showed an increase in risk associated with use of OC only in the full-cohort and an association with longer duration of use (*p* trend = 0.001), especially before FFTP (*p* trend = 0.002). In most of the studies, however, differences in type or dosage of OC were not described. According to the available data, a reliable statement on possible effects of different formulations can therefore not be made.

Overall, data on the risk of OC use in *BRCA* mutation carriers are limited. Almost all of the available studies are retrospective and especially for *BRCA2* mutation carriers, study populations were often small. Apart from known problems of retrospective studies as recruitment bias and survival bias, the here discussed studies have multiple limitations. Study designs were different and genetic testing for controls not always complete. Partly, women with risk-reducing bilateral oophorectomy or prophylactic mastectomy were included [5, 11]. In some of the studies, specific ethnic subgroups were overrepresented [5, 15].

Risk of breast cancer is high in *BRCA1/2* mutation carriers, and one or several modifiers with a small effect that is multiplicative can have a significant impact in a high-risk subgroup. This was postulated for low penetrant genetic variants in the past [36]. The only prospective study by Schrijver et al. [35] showed heterogenous results. Being one of the biggest cohort studies to date, the power to stratify for age groups of exposure and for age of onset of breast cancer was still too low. A relevant risk elevating effect of OC use for breast cancer is so far not proven but especially in the specific age group of young *BRCA1/2* mutation carriers before the age of 40 years is still possible. Larger prospective cohort studies with longer follow-up and power to stratify for age groups of exposure and for age of onset of breast cancer are therefore urgently needed.

When illuminating the effects and safety of OC in *BRCA1/2* mutation carriers, many different aspects must be taken into consideration. The protective effect for ovarian cancer is opposed to the possible increase in breast cancer risk. After having had a risk-reducing mastectomy or bilateral salpingo-oophorectomy (BSO) in the past, separate evaluation of the modifying effect of OC use is helpful for further individual decisions. But giving clinical recommendations are complicated by the fact that potential interaction between external hormonal exposure and other modifying genetic or non-genetic risk factors and its influence on cancer risk are not yet clear.

As mentioned above, data are limited in number as well as study types. Prospective randomised studies are not likely to be conducted due to ethical reasons as well as safety aspects. Therefore, clinical registries will be needed for further evaluation. Additionally, linkage with national registries is necessary to improve the follow-up of the participants. The prospective cohort study of the German Consortium Hereditary Breast and Ovarian Cancer (GC-HBOC) is conducting the HerediCaRe registry, which is part of the International *BRCA1* and *BRCA2* Carrier Cohort Study (IBCCS) [37], one of the largest registries for hereditary breast and ovarian cancer. With the result of several prospective cohort studies, finally a level of evidence (LOE) of 2a according to Oxford nomenclature will be achievable. Until then, carriers of mutations in the genes *BRCA1* and *BRCA2* as well as in other less frequent cancer genes should be taken care of under trial conditions. This is very important since uptake of risk-reducing BSO is high and even in well-conducted prospective cohort studies it is going to be difficult to accumulate sufficient follow-up time to definitely answer the question of ovarian cancer risk modulation in carriers by OC use.

Oral contraception leads to a risk reduction of ovarian cancer also in *BRCA1/2* mutation carriers. An increase in breast cancer risk due to OC cannot be excluded. Even though ovarian cancer risk is lower with OC use, it stays elevated and timely risk-reducing BSO is recommended at the age of 40 years for *BRCA1* and at the age of 45 years for *BRCA2* mutation carriers. Adequate hormonal therapy to avoid postmenopausal symptoms and chronic diseases resulting from low oestrogen levels such as osteoporosis and myocardial infarction is recommended until the age of 50 years. Women with *BRCA1/2* mutation who consider OC use have to be informed that this method may lead to an increase in breast cancer risk. OC may be used for contraception in women with *BRCA1/2* mutation, but they have to be informed about alternative methods. When no contraception is needed, OCs should not be used for the prevention of ovarian cancer in this population to avoid a possible increase in breast cancer risk.

## Data Availability

Open Access funding enabled and organized by Projekt DEAL
